# Scientometric study of academic publications on antioxidative herbal medicines in type 2 diabetes mellitus

**DOI:** 10.1186/s40200-016-0273-3

**Published:** 2016-10-21

**Authors:** Ozra Tabatabaei-Malazy, Amir Ramezani, Rasha Atlasi, Bagher Larijani, Mohammad Abdollahi

**Affiliations:** 1 Diabetes Research Center, Endocrinology and Metabolism Clinical Sciences Institute, Tehran University of Medical Sciences, Tehran, Iran; 2 Endocrinology and Metabolism Research Center, Endocrinology and Metabolism Clinical Sciences Institute, Tehran University of Medical Sciences, Tehran, Iran; 3 School of Health Management and Information Sciences, Iran University of Medical Sciences, Tehran, Iran; 4 EBM Group, Endocrinology and Metabolism Research Center, Endocrinology and Metabolism Clinical Sciences Institute, Tehran University of Medical Sciences, Tehran, Iran; 5 Department of Toxicology and Pharmacology, Faculty of Pharmacy, and Pharmaceutical Sciences Research Center, Tehran University of Medical Sciences, Tehran, Iran

**Keywords:** Herbal medicine, Antioxidative, Diabetes mellitus, Scientometric analysis

## Abstract

**Background:**

Scientometric analysis is increasingly used for research assessment. We aimed to perform a scientometric analysis of research productivity in field of antioxidative hypoglycemic herbal medicine and diabetes.

**Methods:**

Some of search terms were “type 2 diabetes”, “antioxidant”, “herb”, “phytotherapy”, “ethnopharmacology”, “Chinese medicine”, “traditional medicine”, in Scopus web databases until January 2015, and limited to human. The collected data were used to generate the specific features such as publication year, main journal in the field, citation, subject area, and co-authorship network of authors and institutes. Data was analyzed using analysis tools provided by Scopus database, SPSS version 11 and VOSviewer software.

**Results:**

Overall, 468 studies were related to this topic in human. The number of publications in the field showed an increasing trend. Majority of the published papers were original articles (71 %) and the most productive year was 2013. Top subject areas were medicine followed by drug. The first productive country was the US. The documents were cited totally 10724 times with average citation/article 22.91, and h-index 55. The highest cited article was a systematic review study, and top source was “Journal of Ethnopharmacology”. The highest international collaboration was with the US. Top authors and institutes in the co-authorship network assessment were from Iran.

**Conclusions:**

A promising scientific productivity is shown in the studied field world wide. This study provided practical information to researchers who look for studies with potentially highly citation, and also would be helpful for researchers to conduct better researches that eventually could lead to more publications in this field.

## Background

The prevalence of Diabetes Mellitus (DM), as a major health problem, is increasing worldwide. International Diabetes Federation (IDF) in its last report stated that the number of diabetic patients will reach from 415 million in 2015 to 642 million in 2040 [1]. Many novel synthetic drugs have been developed in recent years for treatment of DM; however, their usage is limited due to adverse effects, high cost as well as limited accessibility in many countries [2]. These facts make it logic to consider alternative treatments such as herbal medicine for the management of diabetes. Evidences in both developing and developed countries have shown that this kind of therapy has increasing popularity and usage [3–6]. However, there has been no systematic analysis of scientific trends in this field. The bibliometric method as a reliable and practical method can measure, evaluate, and analyze the scientific advance and also determine the current research directions in a specific field [7]. Many indicators are suggested as an index for evaluation of scientific research; however main focuses are on numbers of published papers in a specific field and numbers of their citation [7]. Some of other important indicators are collaboration in research conduct and scientific publications or collaboration in research centers [8, 9]. However, to design a good preventive program and also to determine the safety and efficacy of herbal medicine in the management of type 2 DM (T2DM), scientific evidences provided by scientific papers and reports are needed [2, 4, 6, 10]. Since, oxidative stress is known as the main underlying pathology of diabetes and its complications [11–13], the scientometric analysis of academic publications on antioxidative hypoglycemic herbal medicines would be important not only to the scientific community for recognition of trends in herbal medicine in order to design appropriate prevention programs, but also for researchers to recognize the highly cited studies in order to conduct studies with strong evidences. Considering above points, we aimed to perform a scientometric analysis of scholarly products in antioxidative herbal medicines used for management of T2DM.

## Methods

### Data source

A descriptive bibliometric study of scholarly published articles covering the role of antioxidative hypoglycemic herbal medicine in the management of T2DM was conducted. For this mention the Scopus web databases available at http://www.scopus.com/ was chosen. The reasons for choosing this database includes: high multidisciplinary coverage, especially in health and biomedicine disciplines, high coverage of citation reports, and availability of different analysis tools [8, 9].

### Search strategies

To find relevant studies, we chose the best and most related key words according to the list of Medical Subject Headings (MeSH) provided by the National Library of Medicine (NLM)/PubMed. Our search terms were “type 2 diabetes”, “NIDDM”, “hyperglycemia”, “glucose”, “antioxidant”, “antioxidative”, “plant*”, “herb*”, “component”, “phytotherapy”, “ethnopharmacology”, “naturopathy”, “Chinese medicine”, “herbal medicine”, and “traditional medicine”. The ‘*’ is a wildcard that can take any value. All relevant available academic studies, including review articles, original articles, case reports, conference abstracts, and letters, conducted to assess the effects of antioxidative hypoglycemic herbal medicine in the management of T2DM in human and published before January 2015 were included in the analysis. After assessment the title and abstract of enrolled papers, and exclusion duplicated articles, studies that conducted in children, pregnant women, patients with type 1 DM, or animal studies were also excluded. No language restriction was used. Finally, 468 documents remained for analysis.

### Data analysis

The impact factors (IF) of the journals were retrieved from the Journal Citation Report (JCR) available at http://scientific.thomson.com/products/jcr. While the impact of a journal is often judged by its IF, the numbers of a given article cited by other investigators reflect the importance of that paper [14]. IF as a quantitative indicator is used to assess, compare, and rank the scientific publications in different scientific areas. We investigated SCImago journal rank (SJR) in addition to IF. SJR in fact is a measure of scientific influence of scholarly journals that not only includes the number of citations received by a journal, similar to IF, but also accounts for the importance of the journals where such citations come from. IF and SJR are indicators which used to estimate above mentions for extracting data of ISI or Scopus web databases, respectively.

The *h*-Index of authors which is based on the highest number of included papers having at least the same number of citations was extracted from Scopus. The *h*-graph displays the *h*-index for a single or multiple authors, or a group of selected documents. The *h*-graph for a group of selected documents measures the impact of a set of documents and shows the number of citations per document [15]. The h-index could be used as a measure of research performance quality [15, 16]. Further characteristics of the collected data, including: publication year, the main source (journal) in the field, author’s name and, affiliation, geographical distribution (country/territory), document’s type and language, subject area, and citations were retrieved from Scopus and analyzed using the ‘Analyze search results’ function provided by the Scopus database.

The 468 target results with all available information retrieved from the Scopus database in CSV format. Then the CSV file was converted to Web of Science (ISI WOS) plain text (wos.txt) through scopus.exe and scop2wos.exe tools (http://www.leydesdorff.net/scopus_ovl/). Intcoll software (http://www.leydesdorff.net/software/intc) was used for assessment of studies with international collaboration. To do this, the plain text file was first imported to Intcoll and then results were imported to Pajek software (http://pajek.imfm.si) and visualized. The Scopus CSV file was also imported to VOSviewer free software (www.vosviewer.com/) to visualize the co-authorship network of authors and institutes in the field. The two views (label view and density view) out of four views (label views, density views, cluster density view and scattered view) of VOSviewer were applied in this research. The label view uses the matrix of terms “co-occurrence frequencies” and is particularly useful for a detailed examination of a map. The density view is particularly useful to have an overview of the general structure of a map in order to draw attention to its most important areas. Spearman’s test was used to determine the correlation between number of documents published by a country and total and average citations to them using the SPSS (version 11) (SPSS Inc., Chicago, IL, US).

## Results

**Fig. 1:**
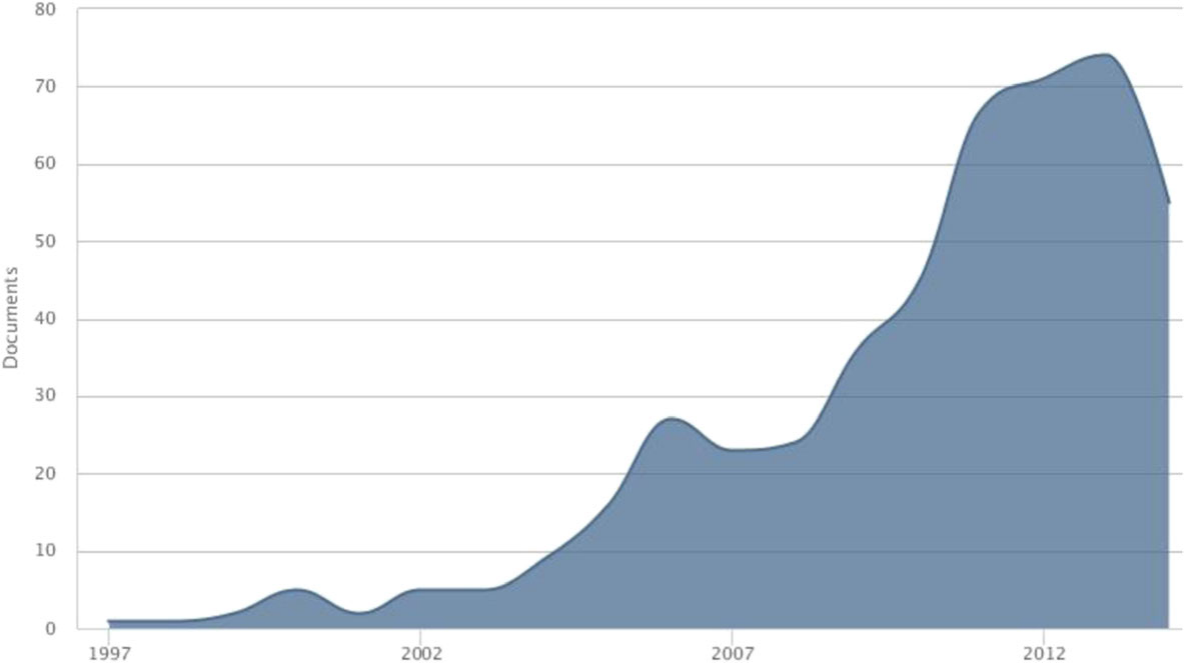
Time-trend in number of published documents in studied field

The trend of annual publications over time is depicted in Fig. 1. The most productive year was 2013 with 74 published documents (15.82 %). The weakest results came from 1997 and 1998 in which just one document per year was published. It is shown a pike in number of published articles in 2006 and 2011 compared to previous years.

Among our analyzed paper, 328 were original articles (70.1 %), 116 were review articles (24.8 %), and 24 (5.1 %) were classified in a miscellaneous group.

Among subject areas of the documents, the top subject area was medicine with 228 documents (61.5 %) followed by pharmacology/toxicology/pharmaceutics (167 documents, 35.7 %) and biochemistry/genetics/molecular biology (157 documents, 33.5 %).

The most of the documents were published in English (93.6 %).

When retrieved data were analyzed by the country, the United States with 84 documents (17.95 %), India with 51 (10.9 %), China with 48 (10.25 %), and Iran with 46 documents (9.82 %) were the most productive countries in regard to number of published documents. According to the number of citations, the United States with 3452 citations (30.21 %) was ranked first and the United Kingdom with 1019 citations, Japan with 857 and India with 829 citations obtained the subsequent positions.

When we assessed the collaboration between countries for published documents in the studied field Yemen, Bangladesh, Bulgaria, South Africa, Slovakia, Saudi Arabia, Romania, Portugal, Lebanon, Hong Kong, and Indonesia had no collaboration with other countries. Among countries, the United States had the highest rates of scientific collaborations.

In assessment the changes in the total number of citations in each year, total number of citations for the retrieved papers was 10,724 times at the time of data analysis (May 18th, 2015) with the average citation per article of 22.91. Among articles, 402 (86 %) documents were cited at least once and 66 (14 %) items did not have any citation at all. The highest number of citations was done in 2013 with 2278 citations while in 1997 no citation was occurring.

**Fig. 2:**
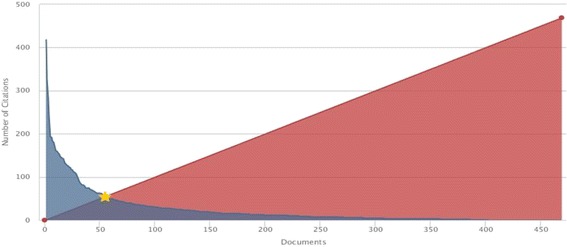
H-graph of published documents in the studied field

The *h*-index for the 468 documents analyzed in this study was 55. This means that from documents considered for calculation of h-index, 55 documents were cited at least 55 times (Fig. 2).

Because of considering articles with >100 citations as highly cited article, there were 29 highly cited articles. The United States, UK, India, and Japan respectively, had the greatest contribution in publishing highly cited articles. Journal of “Diabetes Care”, and “Asia Pacific Journal of Clinical Nutrition” each one with publishing the two out of 29 top cited articles (6.9 %) ranked as the first journals, following by “Journal of Ethnopharmacology”, “Journal of Alternative and Complementary Medicine”, and “Phytotherapy Research” each one with publishing one highly cited article (3.44 %). Of the top ten highly cited articles, four papers were original articles, three papers were review and the last three were conference papers. The United States with four highly cited articles was ranked as the first country having the highest cited papers. South Korea and Australia each one with two highly cited articles ranked second. The journal of “Diabetes Care” with 301 citations per article ranked as the first journal. “Asia Pacific Journal of Clinical Nutrition with 2 highly cited articles, and 145.5 citations per article was the second. General journals with 22 out of 29 top-cited articles published more than 75.86 % of the top-cited articles.

There was a strong positive correlation between citation per top 10 papers and the impact factor of the journals (*r* = 0.81, *P* = 0.015). The highly cited article was a systematic review sudy published in year 2003 in “Diabetes Care” journal (total citation number 418).

We found 2034 authors that published documents in the field of hypoglycemic antioxidative herbal medicine in T2DM. Fallah Huseini, H.” with 14 publications had the highest number of publications in this field. “Haddad, P.S.” with 10 articles, “Larijani, B.”, and “Arnason, J.T.” each one with eight articles, and “Heshmat, R.” with seven articles have published the highest number of articles in this field, respectively.

Based on the above data, of top 10 authors, six authors were from Canada and 4 authors were from Iran. “Tehran University of Medical Sciences”, “Iranian Academic Center for Education, Culture and Research”, “Montreal Diabetes Research Center”, and “Universite de Montreal” each one had two documents.

Co-authorship is one of the factors for evaluation of scientific collaboration that makes a social network among researchers. Based on the unit of analysis, this network is divided into 4 types: co-authorship network of authors, co-authorship network of institutes, co-authorship network of countries, and co-authorship network of locations. In this part of study, type 1 (co-authorship network of authors) was assessed. In order to map the co-authorship network of authors using VOSviewer software, minimum number of documents published by an author was considered to be two documents. Out of 2034 authors, 245 authors meet this threshold. After excluding authors without co-authorships (20 authors), 225 authors were remained and analyzed.

**Fig. 3:**
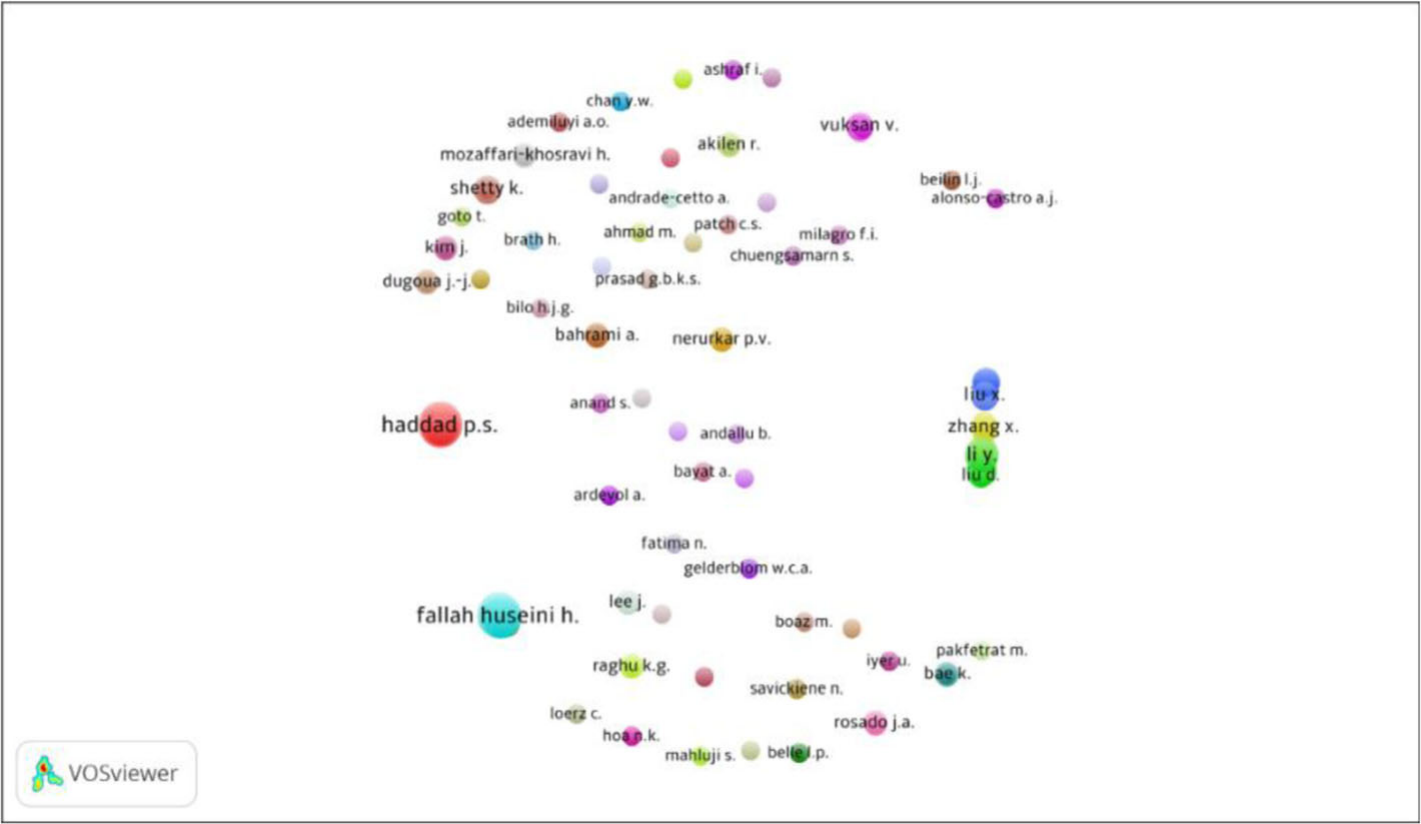
Lable view of co-authorship network of authors for published documents in the studied field

**Fig. 4:**
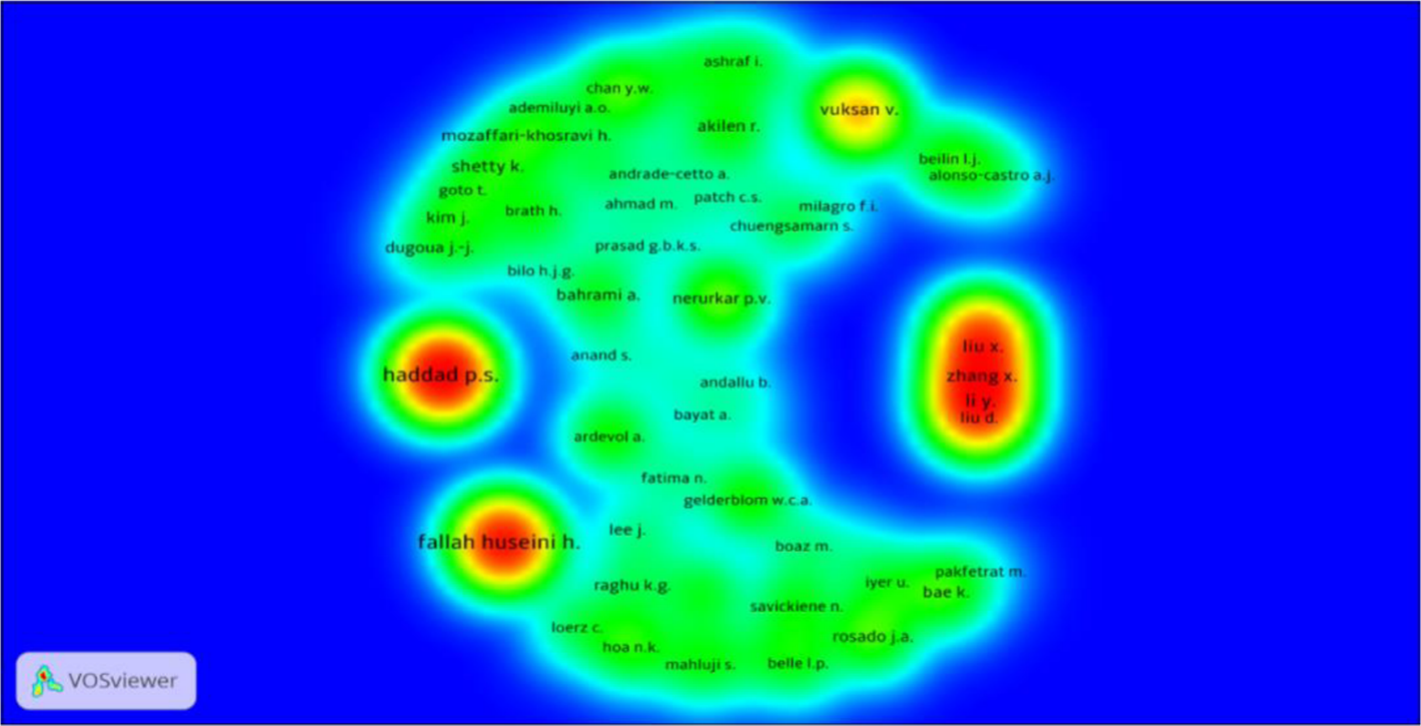
Density view of co-authorship network of authors for the published documents in the studied field

Co-authorship network of authors in the field, in lable and density views are shown in Figs. 3 and 4, respectively.

Cluster analysis of co-authorship network of researchers in the field showed that these clusters included 61 clusters in different colors. Cluster 1 (Red) with Haddad P.S. and Arnason J.T., Cluster 2 (Green) with Li Y. and Zhang X.، cluster 6 (Blue) with Fallah Huseini H., Larijani B.، Heshmat R.، Kianbakht S., and cluster 24 (Brown) with Bahrami A. were the most important clusters. The highest density in the network belonged to Fallah Huseini H., Haddad P.S., Zhang X., Liu X., and Li Y.

Top 10 authors in the field of hypoglycemic and anti-oxidative medicinal plants based on co-authorshipwere Fallah Huseini H. with 11 co-authorships, followed by Haddad P.S. (10), Amason J.T. (8), Heshmat R. (7), Larijani B. and Martineau L.C. each one with 6 co-authorships, Li Y., Kianbakht S., and Currier D. each one with 5 co-authorships, and Schuster D. (4), respectively.

The first ranked institute for publishing the documents was “Tehran University of Medical Sciences” with publishing 20 documents, followed by “Iranian Academic Center for Education, Culture” with 12 published paper and “Universite de Montreal” with 11 articles. In accordance with top countries, the top three institutions’ relations were from Canada, three were from South Korea, and 2 ones were from Iran. Details of these findings are shown in Table 1.

**Table 1:** Names and characteristics of top 10 institutes for the published documents in the studied filed

Rank	Institution Name	Documents (n)	Country
1	Tehran University of Medical Sciences	20	Iran
2	Iranian Academic Center for Education, Culture and Research	12	Iran
3	Universite de Montreal	11	Canada
4	Kyung Hee University	9	South Korea
5	University of Ottawa, Canada	9	Canada
6	Universitat Wien	6	Austria
7	Korea Research Institute of Bioscience and Biotechnology	6	South Korea
8	VA Medical Center	5	United States
9	Chungnam National University	5	South Korea
10	Universite Laval	5	Canada

In order to map the co-authorship network of institutes in the studied field in VOSviewer, minimum number of documents published by an institution were considered to be two documents. Of 1260 institutes 33 ones meet this threshold. After exclusion of further 13 institutes which did not have co-authorships, 20 institutions remained in the final analysis.

Co-authorship network of authors in the field, in density view included 8 clusters in different colors. Based on these analysis, “Endocrinology and Metabolism Research Center, Tehran University of Medical Sciences, Tehran, Iran” with six co-authorships had the highest co-authorships among organizations.

Analysis of publishing sources revealed 463 journals and 5 book series in the field. The “Journal of Ethnopharmacology” with 16 documents ranked the first, followed by “Evidence Based Complementary and Alternative Medicine” with 13 documents and “Phytotherapy Research” with 12 documents (Table 2).

**Table 2:** Characteristics of top 10 sources for the published documents in the studied field

Source Title	Document (*n*)	Total citations to document	Citation per document	Highly cited document (*n*)	Total citation to highly cited document	IF	SJR
Journal of Ethnopharmacology	16	360	22.5	1	159	2.939	1.149
Evidence Based Complementary and Alternative Medicine	13	100	7.69	---	---	2.175	0.42
Phytotherapy Research	12	320	26.66	1	122	2.397	0.82
Diabetes Care	10	846	84.6	2	602	8.570	4.46
Journal of Medicinal Food	9	83	9.2	---	---	1.699	0.62
Journal of Medicinal Plants	8	33	4.125	---	---	---	0.17
Journal of Alternative and Complementary Medicine	7	184	26.28	1	105	1.518	0.48
Molecular Nutrition and Food Research	6	99	16.5	---	---	4.909	1.67
Canadian Journal of Physiology and Pharmacology	6	168	28	---	---	1.546	0.69
Asia Pacific Journal of Clinical Nutrition	6	407	78.83	2	291	---	0.7

*IF* impact factor, *SJR* SCImago journal rank

## Discussion

In the present study, scientometric analysis of research activities on antioxidative hypoglycemic plants in T2DM was carried out. We analyzed 468 scientific products extracted from Scopus web databases. Although, the scholarly literatures that were indexed outside of Scopus web databases were not included in this analysis, it should be mentioned that the Scopus search engine has known as one of the best available tools for analysis and tracking the citations of the published articles [17]. Thus, our study could give a clear and reliable picture about the characteristics of research in antioxidative hypoglycemic plants used for T2DM and published in international journals.

Our findings showed that there is an increasing trend in publishing the papers focusing on antioxidative herbal medicines in T2DM within 1997–2014 despite a temporary decrease in 2014. This observation could be related to some international sanction against Iran. It has been shown that international sanction against Iran negatively affected the works of scientists and researchers in a way that most of them could not publish the results of their research as fast as other countries’ researchers [18].

The majority of published products in this searched field were original articles (>70 %). WHO has recommended the scientific evaluation of effective plants for treatment of metabolic disorders such as diabetes [19]. This suggestion could result in increasing the rate of studies using herbal medicine at different levels ranged from cell-based on clinical trials, as are shown in our results.

The top subject areas of published papers in our analysis were respectively in medicine, and then drug. These figures present the growing rate of evidence based studies in hypoglycemic antioxidative herbal medicine as a new target for management of T2DM [2, 20–23].

The majority of studies were published in scholarly valuable international journals with IF > 2, and SJR > 1. These findings show the worldwide importance of this topic. The “Journal of Ethnopharmacology” that is ranked as the first top journal publishing a high number of papers in this field, belongs to Elsevier publisher, and is indexed in some of the most important citation databases such as ISI Web of Science, Medline, Scopus, EMBASE, BIOSIS, CAB Abstracts, and Chemical Abstracts. Based on IF and SJR values, we can assume that the documents of this field are published in relatively high quality journals. H-index analysis of documents revealed that the highest quality papers are mostly published in the high-impact journals and/or seen by more readers.

When we considered the citation numbers according to published year, we found that highest number of citations was reported in 2013. The reason might be related to increased number of published articles in this year. As expected the lowest number of citations was reported in 1998, and 1997, the years that had the lowest published articles in our topic. Over time increase in the number of citations in the field was in fact an indicator of growing interest of the medical professions to this topic.

After considering the citation report according to the article, the top document was a systematic review study published in “Diabetes Care” journal with a 5-year IF of 8.462 and 418 citation. It is well known that systematic reviews with meta-analysis have the highest level of evidence based [24]. This fact could confirm our findings that highest citations belonged to a systematic review study by scientometric analysis. IF that is a good indicator of the research productivity of the specialty can reflect the importance of the paper with its number [14]. On the other word, journals with high IF are journals with high ranking. The analysis of top-cited articles revealed that most of these articles were published before the year 2008. This might be due to availability of older articles for longer periods compared to more recent published papers.

The majority of the papers were from the United States (84 papers). The other top 3 countries were India (51 papers), China (48 papers), and Iran (46 papers). These figures showed the increasing trend of the published studies in this field, both in developed and developing countries with Iran being the fourth country publishing articles in this field. Based on Iran’s 20 year national vision document, it is predicted that Iran would become the highest developed country in science and technology by 2025 [25]. Based on this, a high rate of published scholarly paper is expected from Iran. Despite international sanction against Iran, we found that Iran was placed within the top 5 countries that published the documents in the studied field according to authors’ affiliation. Three authors of these top 5 authors ranked as the first, the third, and the fifth were Iranian which this fact was in line previous studies [26].

The top international collaboration in the field belonged to the United States. In addition, only 6.4 % of documents did not publish in the English language. The reason might be the widespread use of the English language in scientific productions or due to the fact that the United States was the predominant country producing scientific documents in our topic. Even though, our results showed that 4 authors of the top 10 authors in the studied field based on density and label views of co-authorship network faced by VOSviewer software were Iranian, and the first ranking belonged to Iran. In density view researchers with high scientific relations had closer distance and researchers with lower scientific collaboration had farther distance. Density of any researcher was identified by number of scientific productions, number of neighboring node, and importance of neighboring node. On the other hand, being a researcher at the center of density map, illustrated the importance of that node in the co-authorship network of authors. Also, the range of color from red to blue indicated the weight of higher density to weight of lower density nodes. Fallah Huseini H. (with 11), Heshmat R (with 7) and Larijani B (with 6) allocated as the first, as well as the fourth and the fifth ranking of co-authorships were all Iranian. In addition, when we assessed the institutional co-authorship of the countries participated in publishing papers in our searched field, we found that many institutes were engaged in our topic during 1997–2015. Out of them, the first and the second ranking were belonged to 2 Iranian institutes. The “Endocrinology and Metabolism Research Center of Tehran University of Medical Sciences” had the highest co-authorships among organizations. It is known that co-authorship is one of the most tangible forms of research collaboration [27]. Multiplicity and diversity of authors writing in a specific field led to the formation of a common authorship or co-authorship network that in this network the authors have the correlated entities form the global system of knowledge production [27, 28]. In addition, the best bibliometric indicators to illustrate different patterns of co-authorship of academic disciplines are co-authorship networks [29]. Thus, the number of published articles, and number of their citations can be affected positively by number of related multidisciplinary faculties, research centers, and related specialists, students, and research projects [30].

Our study had some strengths and limitations. First, we focused on specific subjects on scientific productions in diabetes’ field. Second, we used Scopus web database that has a high coverage in different branches of science. Third, we assessed the worldwide trends of scholarly articles production concomitant with international collaboration and co-authorship network of authors and institutions. The first limitation of this study was exclusion of non-Scopus journals. Notably, we did not intend to ignore there are some highly cited scientific publications in these journals. The second limitation was the exclusion of articles published before 1996, due to the creation of Scopus web databases in 1996.

## Conclusions

The results of the present scientometric analysis showed promising productivity of scientific publications in antioxidative hypoglycemic herbal medicines in T2DM, and relatively good face of Iran for scientific productions in this field. This study provided practical information to researchers who look for studies with potentially highly citations, and also would be helpful for researchers to conduct better researches that eventually could lead to more publications in this field.

## Abbreviations

ADA: American diabetes association
DM: Diabetes mellitus
IDF: International Diabetes Federation
IF: Impact factor
JCR: Journal citation report
MeSH: Medical Subject Headings
NLM: National Library of Medicine
SJR: SCImago journal rank
T2DM: Type 2 diabetes mellitus
VOSviewer: Visualizing scientific landscapes
